# Advances in CrRLK1L function in plant cell wall signaling during interaction with the environment and development

**DOI:** 10.1007/s44154-025-00231-y

**Published:** 2025-10-16

**Authors:** George Bawa, Yang Shen, Mingzhe Sun, Xiaoli Sun

**Affiliations:** Crop Stress Molecular Biology Laboratory, College of Agriculture, Heilongjiang Bayi Agricultural University, Daqing, 163319 China

**Keywords:** CrRLK1L, Plant cell wall, Cell wall integrity, Stress tolerance, RLKs

## Abstract

As a barrier between the cell and its environment, the plant cell wall provides structural support during development and stress response. Plants are able to sense their surroundings and adjust their activities accordingly. A crucial mechanism involved in these adaptive changes is the cell wall integrity (CWI) maintenance mechanism, which monitors and maintains the integrity of cell walls via changes in cell and cell wall metabolism without destroying cell wall organization. Different abiotic stresses and changes in plant developmental phases disrupt CWI. However, emerging evidence has demonstrated the initiation of CWI signaling mechanisms as key in promoting plant growth in complex situations. This review discusses recent advances in the *Catharanthus roseus* receptor-like kinase 1-like (CrRLK1L) protein function in plant cell wall signaling during adaptation to changing environments and development. We conclude by highlighting how current spatially resolved transcriptomics may be used to advance the role of CrRLK1L members in plant cell wall signaling during development and stress response.

## Introduction

Plant cell walls not only provide structural support or determine cell shape, but they are also instrumental in plant life, acting as the front line of defense for several environmental stresses, such as drought, salinity, extreme temperatures, light, flooding, etc. (Calderone et al. 2024; Rui and Dinneny 2020; Wang et al. 2021b; Yu et al. 2022; Zou et al. 2024). These stress-related conditions can significantly restrict plant growth by retarding cell extensions (Rui and Dinneny 2020; Zhang et al. 2020a). The dynamic nature of plant cell walls enables cells to expand while providing support to counterattack pressure mounted on the cell wall (Colin et al. 2022; Zhong et al. 2024). Plant cells monitor the integrity of their cell walls, and any disruption or impairment in their normal functioning activates cell wall integrity (CWI) mechanisms (Lorrai et al. 2024; McFarlane 2023; Rui and Dinneny 2020). Cell wall malfunction or impairment results from cell wall damage (CWD), which occurs during exposure to abiotic stresses as well as during development (Bacete et al. 2018; Novaković et al. 2018; Wolf 2017). The effects of these stress conditions, along with changes in cell structure during development, significantly impact CWI signaling. However, growing evidence has shown that a plant's ability to sense and maintain CWI under such conditions is critical for enhanced plant growth and development (Houston et al. 2016; Wolf 2022). Over the years, plant receptor-like kinases (RLKs) have been demonstrated to be involved in several critical aspects of plant development, such as growth, morphogenesis, metabolism, reproduction, and stress responses (Cui et al. 2022; Ou et al. 2021; Sun et al. 2018, 2020, 2016). RLKs in plants consist of several membrane protein families that have cell surface signaling capability (Cui et al. 2022).

Among these RLKs crucial in plant cell wall signaling and sensing is the *Catharanthus roseus* receptor-like kinase1-like (CrRLK1L) protein (Nissen et al. 2016). CrRLK1L is the most highly studied RLK in the context of cell wall signaling (Gao and Li 2022; Nissen et al. 2016; Richter et al. 2017; Solis-Miranda and Quinto 2021). CrRLK1Ls have emerged as essential for CWI signaling during stress and are involved in coordinating growth and development in several plant cell types (Solis-Miranda and Quinto 2021). This review discusses recent advances in CrRLK1L members' role in cell wall signaling and sensing during extreme environmental conditions and development. We begin by discussing the pivotal roles of major CrRLK1Ls in cell wall signaling and sensing, their impact on downstream signal pathways, and the mediation of phytohormone pathways during CWI maintenance. Finally, based on these intriguing insights, we propose future cutting-edge research solutions that could be game-changing for CrRLK1L advancement in the context of cell wall signaling.

## Composition of plant cell wall

**Fig. 1:**
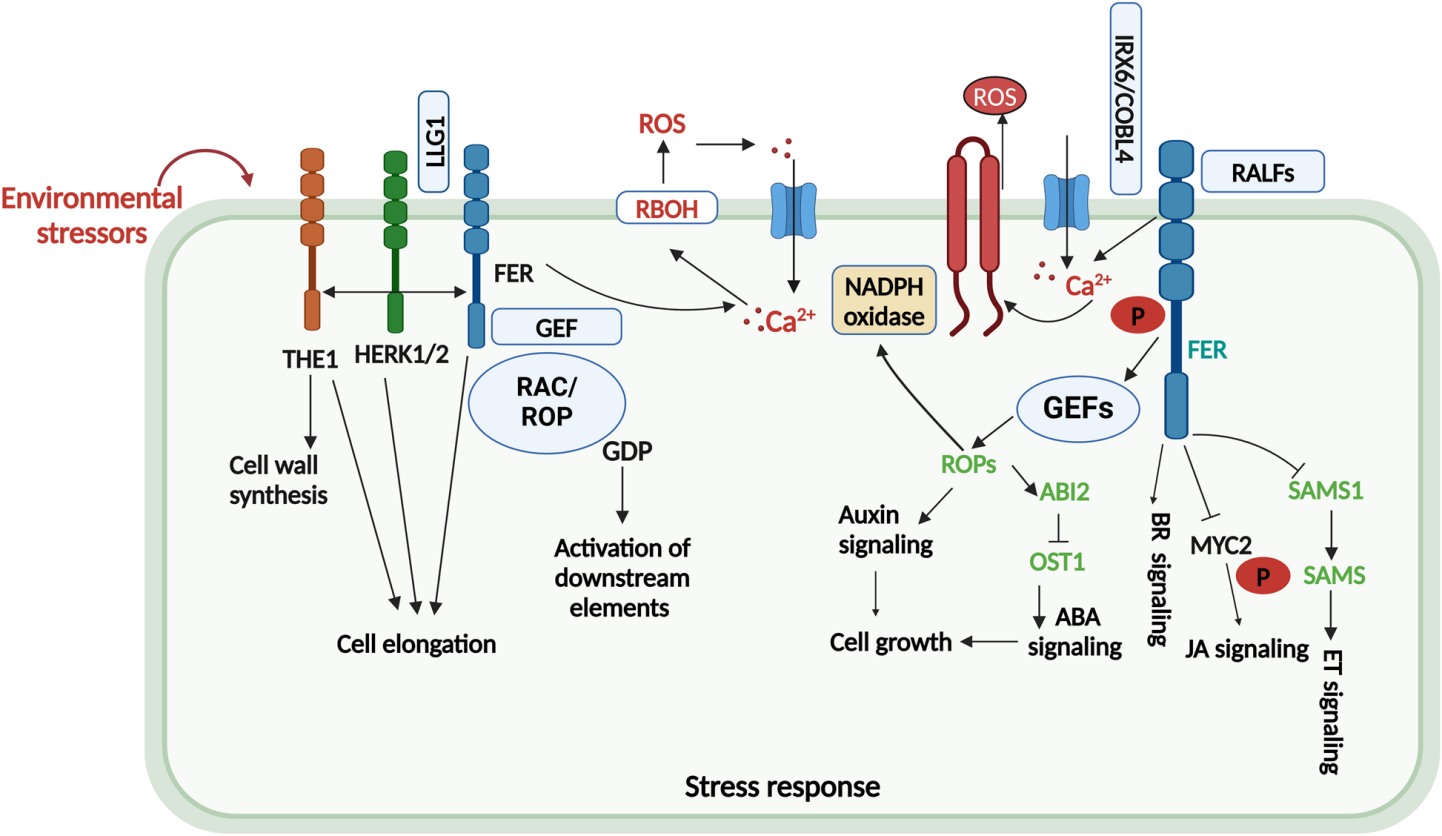
CrRLK1L's involvement in cell wall signaling and cell growth under stress conditions. Cell wall damage resulting from stress signals automatically ignites integrity-sensory elements. FER, THEI, ANX1/2, and HERK1/2 are important for inducing the adaptive responses to cell wall damage. FER possibly functions through a GEF to regulate the activity of GTPase (RAC/ROPs) modules to enhance NADPH oxidase-mediated ROS generation to release downstream elements, thus mediating cell wall signaling and cell growth. In addition, FER interaction with GEF/ROP induces NADPH oxidase-mediated ROS generation in auxin synthesis. COBL4 is involved in cellulose deposition in the secondary cell wall. Also, LLG1 interacts with FER to sense changes in CWI. The FER-RALF module mediates the ABA response via specific GEFs, ROPs, and ABI2 elements. FER negatively regulates SAM synthesis, which represses ethylene production. FER gene expression induces BR signaling. FER also suppresses the JA signaling pathway by phosphorylating and destabilizing the transcription factor MyELOCYTOMATOSIS PROTEINS (MYC2). This highlights the role of the RALF-FER-MYC2 signaling pathway

Plant cells form nanofibrillar walls that are crucial to plant growth and survival (Cosgrove 2024). These walls comprise cellulose microfibrils, hemicellulose, pectins, glycol-proteins, and in some instances, lignin (Hamann 2012), which makes a strong yet extensible wall. These cell walls are classified into primary and secondary cell walls. Primary cell walls are formed during cell division in young growing tissues, while secondary cell walls are formed when cell expansion has ceased. They include thick walls with high mechanical strength (Johnson et al. 2018). Cell walls are dynamic structures that respond to and activate many signaling molecules, which comprise sophisticated control networks that mediate plant responses to environmental stresses and development (Nissen et al. 2016). In the cell wall, cellulose microfibrils form a crucial compartment of the wall. Cellulose microfibrils are synthesized at the cell surface by cellulose synthesis (CesA) complexes (CSCs) and localized in the plasma membrane (PM). They are accumulated in the Golgi and are cross-linked to hemicelluloses and pectin (Pérez García et al. 2011). Although cellulose microfibril linkage is associated with all plant walls, the structure of the matrix varies in diverse plant groups alongside varying developmental levels (primary versus secondary). Cellulose biosynthesis and related genes have been shown to be mediated by several environmental factors, including drought, flood, heat, salinity and cold. (Colin et al. 2022; Novaković et al. 2018). Also, COBRA-LIKE4 (COBL4), a glycosylphosphatidylinositol (GPI)-anchored protein, is required for cellulose deposition in the secondary cell wall during development or under stress conditions (Fu et al. 2024; Qiu et al. 2023) (Fig. 1). The coble4 mutant allele, irregular xylem 6 (IRX6), encodes a member of the COBRA-like family, which is integral to secondary cell wall biosynthesis, especially in xylem tissues (Brown et al. 2005) (Fig. 1). Cosgrove (2005) demonstrated that xyloglucans, a constituent of hemicellulose found in dicot walls, were the key matrix polysaccharides associated with cellulose and microfibrils. Further studies suggest that xyloglucan interacts with cellulose at specific sites targeted by wall-loosening proteins to facilitate expansion (Park and Cosgrove 2015). High-temperature stress has been shown to regulate hemicellulose-cellulose synthesis networks in sweet corn (Suwa et al. 2010). Pectins maintain and control cell wall porosity by forming a hydrated gel phase in which cellulose and non-cellulosic polysaccharides are embedded. Pectin remodeling enzymes, pectin methylesterases, pectin methylesterase inhibitors, and pectin degrading enzymes regulate pectin stiffness and viscosity that mediates wall expansion and cell growth (Cosgrove 2014; Levesque-Tremblay et al. 2015). Pectin modifications in the cell wall have been shown to be essential for dehydration stress (Forand et al. 2022). Though a minor component of the wall—mostly in the primary wall—glycoproteins have a significant role in cell wall properties by remodeling cell wall polysaccharides, polyphenolics, and signaling functions during development and stress-related functions (Novaković et al. 2018), while polyphenolic elements such as lignin and its coupling derivatives are highly expressed in the secondary cell walls during wall acclimation to stress and development (Cesarino 2019; Scheller and Ulvskov 2010).

CWI sensors and mechanosensitive ion channels constantly monitor the mechanical integrity of cell wall structures (Basu and Haswell 2017; Hamann 2014). CWI sensors are involved in maintaining wall strength and expansion during turgor-driven growth. Besides sensing alterations in the physical properties of the cell walls during growth (Gonneau et al. 2018), CWI sensors sense CWD to the wall polysaccharides (Feng et al. 2018) induced by abiotic stresses, which activate wall modifications to mediate the integrity of the wall (Hématy et al. 2007). Though the specific signal mechanisms remain poorly understood, the findings highlight the key functions of these cell walls in plant stress responses and growth regulation. Despite our understanding of the functions of these cell wall components in the last few years, this research area continues to evolve, and several questions continue to emerge regarding how cell wall remodeling in plant stress tolerance impacts wall integrity. For example, recent findings suggest a function of cell wall identity determination analogous to extracellular matrix signaling in animals (Huerta-López et al. 2024; Wolf 2022). Thus, it is anticipated that future studies will possibly be able to follow up on such interesting results.

## CrRLK1Ls and cell wall integrity signaling

The CrRLK1L family, named after the first member identified from *C. roseus* cell cultures (Schulze-Muth et al. 1996), has been demonstrated to be involved in sensing changes at the cell wall and transmitting such information to activate cellular responses during plant growth and development and response to environmental stimuli (Boisson-Dernier et al. 2011; Zhang et al. 2020b). Members of CrRLK1L have been characterized and named in many plant species (Franck et al. 2018), and its role in cell wall homeostasis, integrity maintenance, and growth control has been demonstrated by several studies (Nissen et al. 2016). The model plant *Arabidopsis thaliana* (Arabidopsis) has 17 CrRLKI1L subfamily members (Hématy and Höfte 2008), among which 8 have been well characterized to function in cell wall remodeling (Franck et al. 2018; Gandhi and Oelmüller 2023; Lindner et al. 2012; Nissen et al. 2016). The CrRLK1L subfamily of proteins has two extracellular malectin-like (MAL) domains that possibly bind to cell wall polymers and an intracellular kinase domain that transfers signals through the phosphorylation of intracellular substrates (Franck et al. 2018). This section discusses advances in the major CrRLK1L members involved in CWI signaling during extreme environmental conditions and development (Fig. 1 and Table 1).

**Table 1:** CrRLK1Ls implicated in plant cell wall signalling

RLK family name	Subfamily	Expression	Function in cell wall context	Location	Downstream events	References
CrRLK1L	FER	All tissues except matured pollen	RALF receptor, CWI maintenance	PM	GTPass, ROP, GEF1, ROS, Ca^2+^	Feng et al. (2018) Dong et al. (2019); Guo et al. (2009a); Liu et al. (2024c); Zhao et al. (2021).Duan et al. (2010); Dünser et al. (2019)
	ERU/CAP1	Pollen/Root hair	RALF receptor, CWI maintenance	Tonoplast	ROS	Schoenaers et al. (2018)
	THE1/HERK1/2	All vegetative tissues; high in elongating tissues	RALF receptor, CWI maintenance	PM	ROS, Ca^2+^, MAPK6, GEF4	Denness et al. (2011); Gigli-Bisceglia et al. (2020); Gonneau et al. (2018); Guo et al. (2009a); Hématy et al. (2007)
	ANXUR1/2	Pollen	CWI maintenance, RALF receptor	PM	ROS, RBOH, MPKs kinase, MARIS	Boisson-Dernier et al. (2011); Boisson-Dernier et al. (2013); Boisson-Dernier et al. (2009); Miyazaki et al. (2009)
	BUPS1/2	Pollen	RALF receptor, CWI maintenance	PM	ROP, ROS	Feng et al. (2019)
	HERK1/ CVY1/ANJ-FER	Pollen	RALF receptor, CWI maintenance	PM	ROS	Galindo‐Trigo et al*.* (2020).Lan et al. (2023)

### FERONIA

**Fig. 2:**
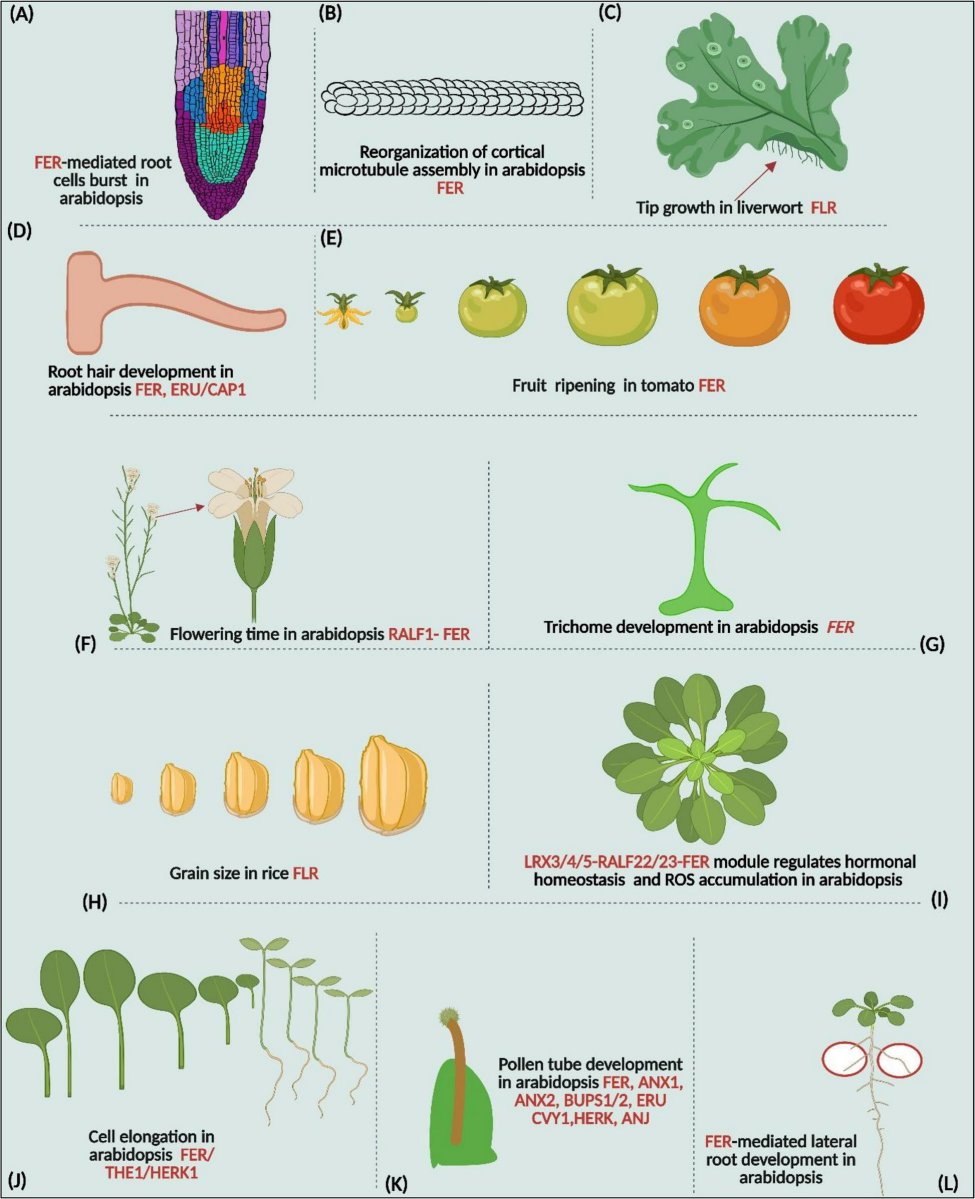
CrRLK1L members function in cell wall signaling and cell growth. **A **FER-mediated root cells burst in Arabidopsis (CWI context) (Feng et al. 2018). **B **FER-mediated reorganization of cortical microtubule assembly in Arabidopsis (CWI context)(Liu et al. 2024c). **C **FER signaling in rhizoid growth in the liverwort (CWI context) (Westermann et al. 2019). **D **FER regulation of root hair development in Arabidopsis (CWI context) (Duan et al. 2010; Marzol et al. 2018; Schoenaers et al. 2018; Zhu et al. 2020). **E **FER regulation of fruit ripening in tomato (CWI context) (Jia et al. 2023, 2017; Yin et al. 2018). **F **RALF1-FER regulation of flowering time in Arabidopsis (growth context) (Wang et al. 2020). **G **Mutation of *FER* involvement in regulating trichome development in Arabidopsis (growth context) (Duan et al. 2010). **H **FLR regulation of grain size in rice (growth context) (Wang et al. 2021a). **I **The LRX3/4/5-RALF22/23-FER module regulates hormonal homeostasis and ROS accumulation in Arabidopsis (CWI context) (Zhao et al. 2021). **J **FER/THE1/HERK1 regulation of cell elongation in Arabidopsis (CWI context) (Guo et al. 2009a). **K **FER, ANX1, and ANX2, BUPS1/2, ERU, CVY1 and ANJ regulation of pollen tube development in Arabidopsis (CWI context) (Boisson-Dernier et al. 2013; Escobar-Restrepo et al. 2007; Feng et al. 2019; Galindo‐Trigo et al*.*, 2020; Lan et al. 2023; Lassig et al. 2014). **L **FER-mediated lateral root development in Arabidopsis (growth context) (Dong et al. 2019)

Feronia (FER) is part of the signaling heterocomplexes in the PM that regulate signal perception. FER, the most studied member of the CrRLK1L family (Fig. 2) (Huang et al. 2024; Liu et al. 2024b, 2023a, 2024c; Nissen et al. 2016), was first identified as a regulator in male–female communication during pollen tube reception (Escobar-Restrepo et al. 2007). With time, its function in plant life during growth, development, reproductive function, hormone signaling, wall integrity perception, and stress responses has been demonstrated in many species (Fig. 1) (Kong et al. 2023; Li et al. 2016b; Liu et al. 2024c; Nissen et al. 2016; Voxeur and Höfte 2016).

For example, FER participates in cell wall architecture maintenance during salt stress responses (Liu et al. 2024b, 2023a). Feng et al. (2018) demonstrated that FER is involved in maintaining CWI in response to salt stress through pectin binding and the activation of Ca^2+^ transients required for the root cell's response to salinity (Fig. 2A). In the study, mutation of *FER* decreased salt-activated Ca^2+^ influx in the root epidermis and increased sensitivity to increased salt levels. It would be interesting to elucidate how salinity affects cell wall chemistry to discover the molecular underpinnings of how FER senses signals from excess salt. The COMPANION OF CELLULOSE SYNTHASES (CC) is essential for reorganizing cortical microtubule activity during the plant response to stress. To investigate whether FER has a similar function in mediating cortical microtubules in plant response to stress, Liu et al. (2024c) investigated FER function on CC activity under salt stress. They found that FER phosphorylates CC1 to regulate CC1 trafficking and cortical microtubule assembly in response to high salinity, which establishes an important link between CWI and intracellular cell wall signaling, thus highlighting how plants maintain CWI in response to salinity stress (Fig. 2B). However, it would be interesting to discover the main elements linking FER to microtubules to help expand our understanding of the relationship between FER and microtubule regulation of CWI response to high salinity. Tip-growing plant cells require tight monitoring by CWI mechanisms. A study by Westermann et al. (2019) reported FER regulation of CWI in rhizoid growth in the *Marchantia polymorpha* (liverwort). They showed that MpMARIS (MpMRI), required for CWI signaling during rhizoid growth, is dependent on MpFERONIA (MpFER) (Fig. 2C). FER has been shown to be essential for CWI sensing in the course of pollen tube development. FER is required for effective rupture of the pollen tube tip and release of spermatic nuclei in the embryo sac of Arabidopsis (Huck et al. 2003; Rotman et al. 2003). In the ovule, *FER* is commonly expressed in the synergid cells and localized at the filiform apparatus to sense the arrival of the pollen tube in order to activate the release of its sperm cells (Escobar-Restrepo et al. 2007). Root hairs play a role in the plant's response to stress conditions by altering various developmental patterns. Many studies have demonstrated FER mediation of CWI signaling in root hair development in Arabidopsis (Fig. 2D) (Duan et al. 2010; Marzol et al. 2018; Zhu et al. 2020). A FER homologue, ERULUS/[Ca^2+^]_cyt_-ASSOCIATED PROTEIN KINASE 1 (CAP1), has been reported to localize at the apical root hair and regulate cell wall composition via pectin methylesterases and phosphorylation of FER (Schoenaers et al. 2018). LORELEI-LIKE GPI AP1 (LLG1), a co-receptor for FER, interacts with FER and is required for FER localization and signaling in the root (Feng et al. 2018; Li et al. 2015) (Fig. 1). Fruit development and ripening are essential biological processes in plant life (Seymour et al. 2013). FER has been shown to participate in the regulation of CWI signaling in tomatoes (Jia et al. 2023, 2017; Yin et al. 2018) by regulating important biochemical metabolisms such as color, firmness, sugar and acids. (Fig. 2E). The RAPID ALKALINIZATION FACTORS 1 (RALF1)-FER module has been shown to regulate cell growth during the flowering period in Arabidopsis (Fig. 2F) (Wang et al. 2020). Mutation of *FER* is involved in cell growth in trichome development in Arabidopsis (Fig. 2G)(Duan et al. 2010). In addition, FERONNIA-like receptor (FLR) members have been determined to be essential in plant development under stress conditions (Li et al. 2016a). In rice, FLR has been demonstrated to regulate cell growth during grain development (Fig. 2H) (Wang et al. 2021a). FER interaction with leucine-rich-repeat extensions (LRXs) has been documented as a major step in detecting CWI maintenance (Zhao et al. 2018). For example, FER, along with LRX3/4/5 and RALFs, is involved in causing changes in cell wall acidification, cell expansion, and loosening in Arabidopsis root under salt stress (Fig. 2I) (Dünser et al. 2019; Zhao et al. 2018). One interpretation of these data is that the FER-modulated perception of CWI possibly requires the support of the LRX3/4/5-RALFs regulatory module.

FER comprises two malectin domains, which bind directly with de-methyl esterified HG in vitro (Feng et al. 2018; Lin et al. 2022). The extracellular MAL domain A binds to cell wall pectin, facilitating FER linkage with the cell wall to mediate pavement cell morphogenesis (Lin et al. 2022). This suggests that FER possibly senses alterations in the cell wall using its extracellular domain and later transfers the cell wall signals to interior cells using its cytoplasmic kinase domain. Several lines of evidence point to the involvement of the nicotinamide adenine dinucleotide phosphate (NADPH) oxidases, Rho/Ras of plants (ROP/RAC)-guanine exchange factors (GEF), and ROP GTPases in CrRLK1Ls downstream signaling events (Duan et al. 2010; Li et al. 2022; Liu et al. 2024a; Smokvarska et al. 2023). Accumulated evidence has implicated FER in reactive oxygen species (ROS) signaling of CrRLK1L downstream events. For example, several studies have demonstrated FER's role in ROS accumulation in various plant organs (Smokvarska et al. 2023). Duan et al. (2010) demonstrated that FER coordinates with ROPGEFs to activate RAC/ROP GTPases to trigger the accumulation of ROS through NADPH oxidases. Ligands of FER, including RALF peptides RALF1, RALF23, and RALF33, may trigger ROS accumulation in plant tissues (Liu et al. 2024a). RALF1 has been demonstrated to trigger ROS accumulation through FER, RAC/ROP, and NADPH oxidase-regulated pathways in root development (Li et al. 2022; Liu et al. 2024a). In rice, an NADPH oxidase has been reported as a direct interactor of a RAC/ROP protein (Wong et al. 2007), which has been confirmed in Arabidopsis by characterizing ROP11 as an activating agent of the NADPH oxidase in root hairs (Yan et al. 2016). However, the correlation between these signals warrants further investigation.

### THESUS1

THESUS1 (THE1), a member of the CrRLK1L family, is involved in CWI sensing, cell expansion, and mechanical stress responses upon cellulose impairment (Gigli-Bisceglia et al. 2018; Li et al. 2016b). *The1* was discovered during a screening for suppressors that reduced the short hypocotyl phenotype of dark-grown, cellulose-deficient mutant *procuste1-1* (*prc1-1*), which is less effective in the cellulose synthase catalytic subunit CESA6, hence named after the Greek mythical founder-king of Athens who slaughtered the bandit Procrustes (Hématy et al. 2007). The study discovered that THE1 regulates the response of plant cells during cellulose biosynthesis during growth periods. The findings detailed that two *thesus1* alleles, with their mutation positioned in a melectin-like domain, were found to partially suppress the cellulose-deficient mutant *prc1-1* short hypocotyl phenotype when grown in darkness (Hématy et al. 2007). Such mutations somehow maintained the hypocotyl lengths of dwarf *prc1-1* seedlings and increased cell elongation. The loss-of-function alleles resurrected the dwarf phenotype and ectopic lignification of several *cesa* mutants without increased cellulose deficiency, suggesting that the decreased cell elongation is not a result of a decrease in cellulose level but outcomes of cell wall signaling that depends on THE1 (Fig. 2J). In addition, *THE1* is involved in lignin production, resulting from mutations in the cellulose synthase complex (Hématy et al. 2007) or by the cellulose synthesis inhibitor isoxaben (Denness et al. 2011). Priming of isoxaben was shown to activate the production of ROS in wild-type (WT) seedlings but not in *the1* nor in rbo homologous D and F (*rbohD*/*rbohF*) mutants that affect the ROS-producing NADPH oxidase (Denness et al. 2011). These data suggest that during impairment of CWI, THE1 induces a signal transduction pathway, resulting in ROS accumulation, growth impairment, and lignin generation, highlighting regulatory mechanisms that require extensive investigations. Contrary to this impairment of cellular growth in CWD perspectives, *THE1* was shown to be involved in cell elongation during vegetative growth in the company of HERCULES (*HERK) 1* and FER (Fig. 2J), a mechanism possibly dependent on the brassinosteroid (BR) signaling pathway (Guo et al. 2009a, 2009b). However, further studies are needed to confirm the putative function of BR in HERK/THEI/FER mediation of cell elongation.

Metal ions bind to cell wall components and induce cell wall changes to trigger growth responses (Muschitz et al. 2015). To determine whether THE1 functions in inhibiting cell elongation in hypocotyls during metal ion homeostasis, Richter et al. (2017) investigated whether nickel (Ni) induces CWD-specific responses. The results showed that the expression of the downstream gene, extracellular dermal glycoprotein (*EDGP*)*/At5g19110,* was increased in the overexpression and the hypermorphic allele with no response in the loss function allele, *the1-6,* in chemically induced CWD via priming with the cellulose biosynthesis inhibitor isoxaben. After priming with Ni, a decrease in various THE1 alleles was observed, with no significant changes in WT seedlings. The findings suggest that Ni sensitivity depends on CWD-specific gene repression responses. This data establishes a functional link between metal ion stress and the cell wall modification sensors of the CrRLK1L family. Genetic and hormonal analyses have demonstrated that FEI2 (named after the Chinese word for fat) (Xu et al. 2008) functions downstream of THE1 in regulating CWI perception (Engelsdorf et al. 2018). MATING PHEROMONE-INDUCED DEATH1 (MID1)-COMPLEMENTING ACTIVITY 1 (MCA1) is a PM-localized stretch-inducing Ca^2+^ pathway that functions downstream of THE1 in Arabidopsis exposed to hypoosmotic stress (Denness et al. 2011; Nakagawa et al. 2007). Changes in cellulose biosynthesis ignite defense signaling pathways; loss-of-function mutations in *CESA* genes or priming plants with isoxaben activate the assembly of jasmonate and ectopic deposition of lignin (Caño-Delgado et al. 2003; Ellis et al. 2002). Accumulating evidence suggests that such development depends on THE1 (Hamant and Haswell 2017). However, how THE1 transduces cellulose deficiency into defense is insufficiently understood. In addition, the mechanism underlying how THE1 induces signaling by RAC/ROP GTPases via interacting with ROPGEFs is poorly understood. Together, these studies have highlighted THE1's function in cell wall restructuring and remodeling to maintain CWI. However, several areas warrant further research. For example, Gonneau et al. (2018) identified a putative ligand for RALF34, which is required for THE1 regulation of CWI sensing during lateral root development; meanwhile, the signaling pathway activated by RALF34 is poorly understood. Furthermore, it is yet unknown if THE1 binds other ligands, as FER does, and if it interacts with cell wall carbohydrates via its malectin domains.

### ANXUR (ANX) 1/2

ANX1 and ANX2, named after the male consort of FER, are pollen-specific THE/FER family RLKs and are considered the two closest paralogs of FER (Boisson-Dernier et al. 2009; Miyazaki et al. 2009). ANX1 and ANX2 are major players in pollen tube CWI signaling during growth (Fig. 2K) (Boisson-Dernier et al. 2013, 2009; Kessler et al. 2015; Zhu et al. 2018). ANX1 and ANX2 receptors regulate cell wall homeostasis during pollen tube formation by creating heterocomplexes in the cell membrane. The pollen tube ruptures before it reaches the female gametophyte due to growing abnormalities in the double *anx1* and *anx2* (Boisson-Dernier et al. 2013, 2009; Mizuta and Higashiyama 2018; Takeuchi and Higashiyama 2016; Wang et al. 2016). Pollen tube integrity and growth depend on events that constantly cycle to balance the wall synthesis, secretion, assembly, and modification. ANX1 and ANX2 functions possibly depend on their capacity to sense the tip cell wall status to sustain this balance during pollen tube growth in the pistil. The expression of ANXs and FER on the surface of male and female gametophytes suggests that their interaction facilitates fertilization, highlighting a possible interdependency between them (Boisson-Dernier et al. 2011; Miyazaki et al. 2009; Nibau and Cheung 2011). Interaction between ANX1 and ANX2 caused a decrease in fertility and premature pollen tube burst, while overexpression of *ANX1* or *ANX2* decreased pollen tube growth resulting in cell wall accumulation and PM invagination (Boisson-Dernier et al. 2009; Miyazaki et al. 2009). Increased cell thickening followed membrane invagination, which could be consistent with the oversecretion of cell wall material, with cell walls that have limited elongation growth, or a combination of both (Boisson-Dernier et al. 2013). Again, Boisson-Dernier et al. (2013) reported that *FER* and *THEI*, *ANX1,* and *ANX2* enhance the generation of ROS via the pollen-expressed NADPH oxidases *RBOHJ*. In the study, NADPH oxidase-mediated ROS maintains functional calcium dynamics during pollen tube development. However, the underlying regulatory mechanisms remain poorly understood. ROS signaling in CrRLK1Ls downstream has been reported to be crucial in CWI maintenance. In pollen tubes, ROS biosynthesis and calcium signaling have been implicated in the growth regulation and distribution of cell wall materials at the tip (Boisson-Dernier et al. 2013; Lassig et al. 2014; Winship et al. 2011). While a growth burst accompanies cell deposition, the accumulation of calcium in the cytosol occurs alongside during the growth climax; meanwhile, *rbohh rbohj* exhibits more enhanced growth than WT tubes (Lassig et al. 2014). This data indicates that calcium mediation of NADPH oxidase-derived ROS is essential for maintaining tube elongation and exocytosis. ANX1 and ANX2 regulation of pollen tube integrity and FER regulation of pollen tube burst via controlling calcium and ROS activity confirm CrRLK1L signaling in growth control. The data also highlight FER participation in ROS signaling. RALF4 and RALF19 are required for pollen tube development and CWI (Ge et al. 2019). ANX1/2 and BUDDA'S PAPER SEAL (BUPS)1/2 bind to RALF4 and RALF19 to activate signaling processes in plants (Ge et al. 2017). RALF4 binds to LLGs and BUPS1/ANX1 by several regions (Ge et al. 2019). In addition, RALF34 serves as an important signal required for CWI maintenance by competing with RALF4 and RALF19 to enhance the pollen tube's response to the ovule environment enabled by FER (Ge et al. 2017). In addition, Feng et al. (2019) revealed that pollen-specific LLG2 and LLG3 interact with the ANXs/BUPSs to enhance their secretion to the apical PM. LLG2 and LLG3 play a role as co-receptors of ANX/BUPS to sense the RALF4 signal and trigger ROS accumulation to enhance pollen tube development. HERK1 and ANJEA (ANJ) have been shown to be possible determinants of pollen tube reception. In the study, genetic and biochemical analysis showed that HERK1 and ANJ act with FER to regulate female-male gametophyte interaction during plant fertilization (Galindo‐Trigo et al*.*, 2020). In another study, FER/CURVY1 (CVY1)/ANJ/HERK RECEPTOR KINASE1 and cell wall proteins LRX3/4/5 network on the papilla cell surface with autocrine stigmatic RALF1/22/23/33 peptide ligands to regulate pollen tube development (Lan et al. 2023). These data highlight the involvement of CrRLK1L proteins in mediating reproduction in flowering plants. However, further studies should explore how these RLKs integrate pollen-derived signals to promote fertilization control.

In addition, evidence for the involvement of receptor-like cytoplasmic kinase MARIS (MRI) downstream of CrRLK1L-dependent signaling during root tip growth has also been demonstrated (Boisson-Dernier et al. 2015). The study reported the mapping of mutants, which uncovered an R240C nonsynonymous substitution in the initiation loop of a receptor-like cytoplasmic kinase (RLCK), named MRI, which increased fertility resulting from the rescue of *anx1* and *anx2* pollen tube bursting while negatively impacting the WT background. *mri* loss-of-function mutants indicated decreased transmission via the male gametophyte and showed pollen tube burst, indicating that MRI is vital for pollen tube function. Further studies revealed that MRI acts downstream from NADPH oxidases and depends on its later activity to be effective, indicative of an expression of a gain-of-function but not of the WT version that could rescue the fertility of the *rbohh robhj* mutant. In addition, the *mri* loss-of-function mutant showed root hair defects of *fer*, and the MRI gain-of-function mutant was able to rescue the *fer* defect in root hairs, alongside the expression of MRI, which suggests that MRI functions downstream from FER in root hairs, indicating that RLCKs form part of CrRLK1L-mediated signaling in plant cells. These data demonstrate MRI as a positive component of the CWI pathway. Despite these advances, it would be interesting to determine how FER and ANXs function to promote growth and wall integrity, and how FER triggers cell death. Moreover, determining whether the regulatory mechanisms underlying FER and ANXs are related but expressed differently in the female gametophyte versus the pollen tube requires further exploration. Nevertheless, these findings demonstrate that CrRLK1L proteins mediate CWI maintenance and pollen tube wall modifications during fertilization in a cooperative way. However, despite the advances in CrRLK1L family functions in pollen tube wall modifications, not enough is known about the downstream signal transduction pathways of these receptor kinases in pollen tubes.

## CrRLK1Ls and phytohormone pathways in cell wall signaling

Phytohormone signaling networks enable rapid high-amplitude transcriptional reprogramming during plant development or stress response (Cai et al. 2023; Chen et al. 2022; Dubois et al. 2018; Lu et al. 2021; Shen et al. 2025; Sun et al. 2022; Wang et al. 2024). At the genetic level, RLK genes display several expression profiles when responding to many environmental cues and phytohormones, indicating their molecular functional linkage with a broad spectrum of plant cellular signaling events (Chae et al. 2009). For example, recent studies have shown the CrRLK1L member's versatility in cell wall regulation and serving as a signaling node regulating crosstalk among various phytohormones (Fig. 1) (Chaudhary et al. 2025; Ji et al. 2020; Yu et al. 2020). For instance, Bacete et al. (2022) investigated how the CWI maintenance mechanism regulates cell rigidity and turgor alterations in Arabidopsis*.* The study showed that the accumulation of abscisic acid (ABA), phytohormone-regulated turgor pressure, and water-deficient responses depend on the availability of the cell wall. The results also showed that THE1 regulates mechanical features of the cell wall, ABA synthesis, and ABA-dependent processes. In addition, it has been shown through genetic analysis that the HERK/THE1/FER module is involved in cell elongation and interacts with the BR pathway (Guo et al. 2009a). In the study, *FER* gene expression was decreased in the BR-insensitive mutant *bri1-5* and increased in the gain-of-function mutant *bes1-D.* Microarray investigations showed that these RLKs mediated the expression of transcripts related to cell elongation, suggesting the existence of a gene set that is highly mediated by FER and BR to mediate cell elongation (Fig. 2E) (Guo et al. 2009a). Another study investigated the FER maintenance of CWI during BR-induced cell expansion in Arabidopsis. It was shown that BR induces CWD, causing growth changes in fer mutants. In addition, the study demonstrated that BR-signaling, via BIN2-mediated phosphorylation of FER, enables FER biosynthesis at the PM, identifying crucial networks that coordinate hormone signaling with mechanical sensing to decrease cell rupture during hormone-activated cell expansion (Chaudhary et al. 2025). FER decreases root growth through polar auxin transport via mediating microtubule activities and Ca^2+^ signaling (Li et al. 2020). In tomatoes, silencing of *FER* homologues suppressed BR-triggered heat stress tolerance (Yin et al. 2018). In addition, ABA and RALF putatively interact to respond to stress signals (Liu et al. 2024a). Chen et al. (2016) showed that FER kinase activity affected by RALF and ABA plays a pivotal role in heat and cold tolerance as the *fer* mutant showed intensely changed stress tolerance compared with the WT plants. Moreover, Zhao et al. (2021) showed that the LRX3-5-RALF22/23-FER module mediation of plant development and stress tolerance depends on phytohormone (jasmonic acid (JA), salicylic acid (SA) and ABA) signaling.

**Fig. 3:**
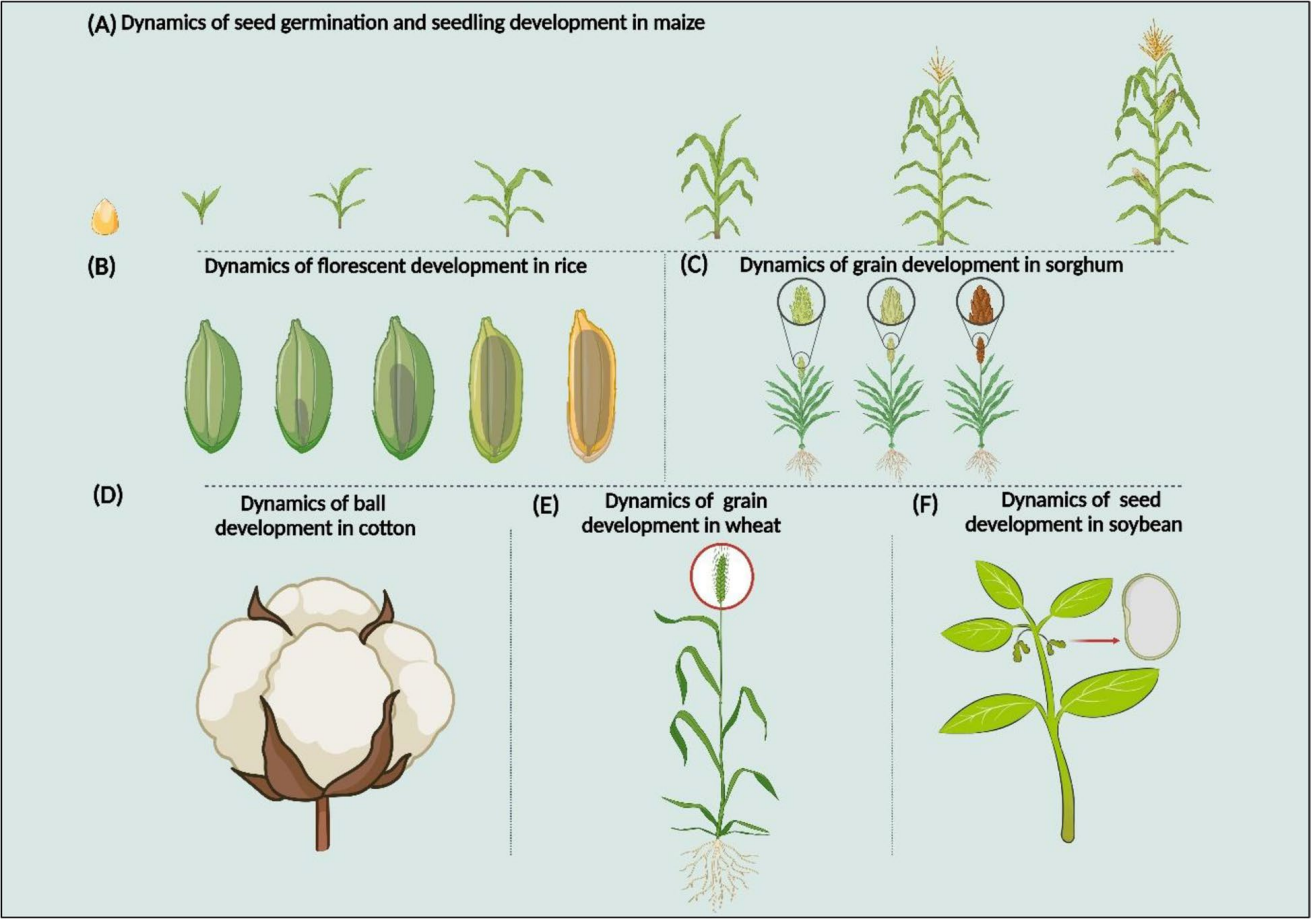
Future applications of spatial transcriptomics in the study of CrRLK1L's role in plant cell wall signaling under stress or non-stress conditions. **A** The dynamics of seed germination and seedling development in maize. **B** The dynamics of fluorescent development in rice. **C** The dynamics of grain development in sorghum. **D** The dynamics of boll development in cotton. **E** The dynamics of grain development in wheat. **F** The dynamics of seed development in soybean

CrRLK1Ls act downstream with phytohormones for efficient activation of stress defense and growth regulation in plants (Fig. 1). For example, FER functions in a ROPGEF1/RAC/ROP2 signaling pathway as a mediator for auxin-regulated root hair development (Duan et al. 2010). In addition, FER mediates the F-actin-dependent polar localization of PIN-FORMED 2 (PIN2) and polar auxin transport, which regulates lateral root development in response to gravity (Fig. 2L) (Dong et al. 2019). Li et al. (2020) further revealed that FER modulates root growth through PIN2 and AUXIN RESISTANT (AUX1)-regulated auxin transport, suggesting a correlation between FER and auxin signaling (Fig. 1). In addition, the mutation in GEF1/4/10 or ROP11/RAC-LIKE GTP BINDING PROTEIN 10 (ARAC10) can imitate *fer-4*, a sensitive mutant of ABA (Yu et al. 2012). Moreover, phytohormones act downstream with cell wall sensors such as leucine-rich repeat extensions LRX3/4/5, peptides RALF22/23 and FER to negatively mediate the levels of JA, SA, and ABA to control plant development in response to salt stress by regulating ROS production (Fig. 1) (Zhao et al. 2021). It has been shown that RAC/ROP phosphorylates and deactivates phosphatase type 2C (PP2C) phosphatase ABA Insensitive 2 (ABI2), a key player in ABA signaling (Nishimura et al. 2010; Yu et al. 2012). In another study, *fer-4* mutants have been shown to be associated with ethylene (ET) synthesis via the actions of S-adenosylmethionine synthetase 1/2 (SAM1 SAM2) as direct interactors of the FER kinase domain. The study also demonstrated how FER impedes SAM1 and SAM2 via phosphorylation, decreasing S-adenosylmethionine production and reducing ET synthesis (Fig. 1) (Mao et al. 2015). The data show a CrRLK1L pathway where one receptor networked with several others to induce many downstream signals. However, studies considering the crosstalk of CrRLK1L signaling with BR, ET, and ABA will be interesting areas to consider. Together, these findings demonstrate that FER functions via ROPGEFs to activate downstream signaling events in phytohormone signaling in plant cell wall function (Fig. 1). Despite these advances, FER crosstalk with hormone pathways is still complicated and offers several arguments that need to be addressed in the future. For example, more investigations are needed on the RALF-FER complex in hormone signaling, stress responses, and crosstalk between stress and phytohormones from the perspective of CWI signaling to fully understand the function of this module. In addition, it is unclear whether and how other CrRLK1Ls mediate hormonal pathways in the context of cell wall homeostasis under stress conditions.

## Conclusion, unresolved questions and the way forward

Despite their existence outside the PM, plant cell walls undergo several modifications to support plant life in many ways. Because cell walls provide support for the cells they encase, monitoring their status during growth, development, or response to stress conditions is essential for plant life (Baez et al. 2022; Cosgrove 2015; Malivert and Hamant 2023; Wolf 2022). Maintaining CWI is important in several biological processes, and the presence of CrRLK1Ls in many plant species indicates that CWI signaling may be present in the entire plant kingdom (Franck et al. 2018; Honkanen et al. 2016). Many studies have demonstrated that CrRLK1Ls function in plant CWI maintenance during growth or response to environmental signals (Table 1 and Fig. 1) (Galindo-Trigo et al. 2016; Wolf 2022; Zhu et al. 2021). These studies have highlighted CrRLK1Ls as critical players in plant development, especially during CWI signaling (Guo et al. 2009a; Nissen et al. 2016; Solis-Miranda and Quinto 2021; Zhu et al. 2021). While these studies have improved our understanding of the CrRLK1L members' function in cell wall homeostasis and biosynthesis, the field continues to develop, and many questions remain unanswered. For instance, CrRLK1Ls are involved in several plant organs and cell types and are required for numerous biological developments. Could the identification or discovery of new peptides or hormone ligands of CrRLK1Ls be beneficial in CWI maintenance? How do CrRLK1Ls sense many RALF peptides to activate response signaling patterns to achieve tissue specificity? How do CrRLK1L members interact with pectins to regulate CWI? How do CrRLK1Ls coordinate with other RLKs to mediate cell wall function? Last but not least, the RALF-CrRLK1L module mediates several agronomic traits. Can the RALF-CrRLK1L signaling function in CWI be used to improve plant agronomic qualities? The question is, what would be the answers to these aforementioned questions? Time will tell. While waiting, we propose compelling research solutions that could redefine or change the face of CrRLK1L's function in cell wall homeostasis and signaling toward enhancing crop improvement.

The accumulated number of studies on CrRLK1L members involvement in cell wall signaling during plant development and response to the environment have significantly focused on genes involved in cell wall metabolism, especially in the model plant Arabidopsis (Boisson-Dernier et al. 2011; Gao and Li 2022; Nissen et al. 2016). However, the molecular mechanisms governing their functions and interactions remain unknown. Assuming that each cell's position within a tissue is crucial for its function, a detailed analysis of tissue spatial gene expression patterns is essential to decipher the role of cell formation in a given tissue, either under stress or during development (Giacomello 2021). Current high-throughput technologies are beginning to redefine our understanding of the regulatory molecular mechanisms surrounding plant development and how they respond to changing environmental conditions. One example is spatial transcriptomics (ST) (Gulati et al. 2025; Rao et al. 2021; Serrano et al. 2024). Compared with single-cell RNA sequencing transcriptomics, which obscures spatial information about cells as a result of protoplast isolation (Shaw et al. 2021; Zheng et al. 2023), ST enables the identification of spatial distributions of RNA transcripts in tissues, not limited by cell wall formation and does not involve protoplast isolation (Zhang et al. 2021). In recent times, ST technologies have become a novel way of generating *in-situ* gene expression information of plant tissues at an unprecedented resolution (Liu et al. 2023c; Rao et al. 2021; Song et al. 2023). ST technologies provide an unbiased picture of tissue spatial composition and have been used to produce tissue atlases, which provide a valuable resource as reference maps (Rao et al. 2021). Additionally, ST data can easily be amended or integrated with other data modalities, providing an expandable framework for insights into tissue organization (Rao et al. 2021). Despite the inability to capture adequate mRNAs, provide spatiotemporal information at high resolution with the best sensitivity (Maynard et al. 2021), or provide information on cell subtypes (Xia et al. 2022), over the years, ST has advanced our understanding of critical aspects of plant biology in model and non-model plants (Giacomello et al. 2017; Lieben 2017; Rao et al. 2021).

Based on the above evidence, we hypothesized that ST could be used to study the role of CrRLK1Ls in cell wall dynamics during growth or response to the environment to reveal several opaque mechanisms in other crops besides Arabidopsis. For example, seed germination and seedling growth are critical stages in the establishment of a maize plant (Liu et al. 2023b; Meng et al. 2022). Genes associated with cell wall metabolism are crucial in seed germination and seedling development (Ma et al. 2017). For instance, loosening the endosperm cell wall is necessary for seed germination and subsequent seedling growth (Farooq et al. 2022; Hurlock et al. 2018). Thus, we hypothesize that ST could be used in maize to facilitate the identification of CrRLK1L's cellular heterogeneity and determination of critical genes that are responsible for cell-type differentiation in maize seed germination and seedling development, which are currently missing at the moment (Fig. 3A). Rice inflorescence development plays a key role in yield formation (Zong et al. 2022). Cell wall composition and biosynthesis genes play a major role in rice development (Lin et al. 2016). This means that using ST, we can identify CrRLK1L member cell types and molecular markers with distinct spatial localization during rice floret development (Fig. 3B), which could provide more avenues for further studies. Stress conditions negatively affect sorghum growth and yield (Abreha et al. 2022). However, cell wall metabolism gene expressions are essential for grain development in sorghum under stress or development (Jain et al. 2008; Rai et al. 2016). Thus, studying the role of CrRLK1L member's function in cell wall context in sorghum using ST can holistically profile the entire cellular and molecular differentiation trajectory of sorghum response to stress signals or growth (Fig. 3C). Boll formation is crucial in determining cotton yield (Muhammad et al. 2025). Several stress conditions severely affect boll formation in cotton; meanwhile, boll development is an essential determinant of cotton yield. However, cell wall biosynthesis genes are very instrumental in ball formation during stress tolerance (Chen et al. 2021; Jin et al. 2024). We assume that research on the role of CrRLK1Ls in cotton ball development using ST to study cell wall compositions enables the identification of cell-specific marker genes and essential developmental trajectories underlying cotton ball formation during development and environment response (Fig. 3D). In wheat, genes associated with cell wall metabolism are required for grain development and stress response (Le Gall et al. 2015; Saulnier et al. 2012). Using ST, we can study CrRLK1L members' function in wheat grain development by enabling the identification of cell-specific marker genes and essential developmental trajectories underlying grain development in wheat under salt stress or non-stress conditions (Fig. 3E). In addition, several environmental and biological processes in soybeans regulate seed development (Hu et al. 2023; Nguyen et al. 2016). However, genes associated with cell wall metabolism are involved in seed formation (Sangi et al. 2019). Meanwhile, the role of the CrRLK1Ls in soybean seed development remains poorly understood. Hence, we assume that ST can be used to discover hidden functions of the CrRLK1Ls in soybean seed development to enable the identification of spatially differentially expressed cell wall regulatory genes controlling seed development in soybeans (Fig. 3F).

## Data Availability

Not applicable.

## Abbreviations

CWI: Cell wall integrity
CrRLK1L: *Catharanthus roseus* receptor-like kinase 1-like
CWD: Cell wall damage
RLKs: Receptor-like kinases
CesA: Cellulose synthesis
CSCs: Cellulose synthesis complexes
PM: Plasma membrane
COBL4: COBRA-LIKE 4
GPI: Glycosylphosphatidylinositol
IRX6: Irregular xylem 6
Arabidopsis: *Arabidopsis* *thaliana*
MAL: Malectin-like
MYC2: Myelocytomatosis proteins
FER: Feronia
CC: Cellulose synthases
MpMRI: MpMARIS
MpFER: MpFERONIA
CAP1: Erulus/[Ca^2+^]_cyt_-associated protein kinase 1
RALF: Rapid alkalinization factors
FLR: Feronnia-like receptor
LRXs: Leucine-rich-repeat extensions
NADPH: Nicotinamide adenine dinucleotide phosphate
ROP/RAC: Rho/Ras of plants
GEF: Guanine exchange factors
ROS: Reactive oxygen species
THE1: Thesus1
*prc1-1*: *procuste1-1*
WT: Wild-type
HERK: HERCULES
BR: Brassinosteroid
Ni: Nickel
FEI2: Named after the Chinese word for fat
MCA1: Mating pheromone induced death1 (mid1)-complementing activity 1
ANX: Anxur
BUPS: Budda's paper seal
LLGs: Lorelei-like gpi-anchored proteins
ANJ: ANJEA
MRI: MARIS
RLCK: Cytoplasmic kinase
ABA: Abscisic acid
JA: Jasmonic acid
SA: Salicylic acid
PIN2: PIN-FORMED 2
AUX1: Auxin resistant
ARAC10: Rac-like gtp binding protein 10
PP2C: Phosphatase type 2C
ABI2: ABA Insensitive 2
ET: Ethylene
SAM1 SAM2: S-adenosylmethionine synthetase 1/2
ST: Spatial transcriptomics
